# The role of co-infections and secondary infections in patients with COVID-19

**DOI:** 10.1186/s41479-021-00083-w

**Published:** 2021-04-25

**Authors:** Charles Feldman, Ronald Anderson

**Affiliations:** 1 Department of Internal Medicine, Faculty of Health Sciences, University of the Witwatersrand Medical School, 7 York Road, Parktown, Johannesburg, 2193 South Africa; 2 Department of Immunology, Faculty of Health Sciences, University of Pretoria, Pretoria, South Africa

**Keywords:** Bacteria, Co-infections, COVID-19, Fungal infections, Outcome, SARS-CoV-2, Severity, Superinfections, Tuberculosis, Viruses

## Abstract

**Background:**

It has been recognised for a considerable time-period, that viral respiratory infections predispose patients to bacterial infections, and that these co-infections have a worse outcome than either infection on its own. However, it is still unclear what exact roles co-infections and/or superinfections play in patients with COVID-19 infection.

**Main body:**

This was an extensive review of the current literature regarding co-infections and superinfections in patients with SARS-CoV-2 infection. The definitions used were those of the Centers for Disease Control and Prevention (US), which defines coinfection as one occurring concurrently with the initial infection, while superinfections are those infections that follow on a previous infection, especially when caused by microorganisms that are resistant, or have become resistant, to the antibiotics used earlier. Some researchers have envisioned three potential scenarios of bacterial/SARS-CoV-2 co-infection; namely, secondary SARS-CoV-2 infection following bacterial infection or colonisation, combined viral/bacterial pneumonia, or secondary bacterial superinfection following SARS-CoV-2. There are a myriad of published articles ranging from letters to the editor to systematic reviews and meta-analyses describing varying ranges of co-infection and/or superinfection in patients with COVID-19. The concomitant infections described included other respiratory viruses, bacteria, including mycobacteria, fungi, as well as other, more unusual, pathogens. However, as will be seen in this review, there is often not a clear distinction made in the literature as to what the authors are referring to, whether true concomitant/co-infections or superinfections. In addition, possible mechanisms of the interactions between viral infections, including SARS-CoV-2, and other infections, particularly bacterial infections are discussed further. Lastly, the impact of these co-infections and superinfections in the severity of COVID-19 infections and their outcome is also described.

**Conclusion:**

The current review describes varying rates of co-infections and/or superinfections in patients with COVID-19 infections, although often a clear distinction between the two is not clear in the literature. When they occur, these infections appear to be associated with both severity of COVID-19 as well as poorer outcomes.

## Introduction

It has been recognised for a considerable time-period, that viral respiratory infections predispose patients to bacterial infections, and that these co-infections have a worse outcome than that of either infection on its own [1]. Best studied in this regard has been influenza virus infection, with the documentation, in several epidemiological and microbiological studies, that most of the deaths occurring during the 1918–9 influenza pandemic were due to secondary bacterial infections, rather than the effects of an inherently hypervirulent virus causing a rapidly progressive, fatal pneumonitis [2–4]. There is similar, although less substantial data, from the 1957 and 1968 influenza pandemics [3]. These factors are said to be important not only during an influenza epidemic/pandemic, with regard to the diagnosis, prevention, and treatment of these bacterial infections, but also in the planning for pandemic preparedness, with the need for stockpiling of antibiotics and vaccines that are active against bacterial pathogens [3, 4]. It has even been suggested that in the setting of influenza community-acquired pneumonia (CAP), empiric antibiotic treatment should be initiated at the same time for bacterial CAP and that this can be de-escalated or discontinued 48–72 h later, especially if no bacterial co-pathogens are recognized [1, 5]. During the H1N1 pandemic influenza, more recently, several studies documented that secondary bacterial infections occur quite frequently, involving other common respiratory viruses and bacteria, and reported that the severity of the influenza, need for intensive care unit admission and mortality of these cases was high [6, 7].

## Coronavirus infections

Coronaviruses have been known to be important human pathogens and relatively common causes of both upper respiratory infections in adults and severe respiratory infections in both adults and children [8]. Severe pneumonia has been associated with outbreaks of coronavirus infection, notably severe acute respiratory syndrome (SARS), and Middle East respiratory syndrome (MERS) [9] and most recently the novel coronavirus, named 2019-nCoV initially, now SARS-CoV-2, that arose in Wuhan in China and which causes severe COVID-19 pneumonia [10–13]. Either these initial studies did not report on the occurrence of co-infections or secondary infections, noted no such infections, or noted a very low rate [10–14].

However, based on the experiences with other viral infections, questions soon began to be asked as to whether this novel coronavirus could be associated with co-pathogens [14, 15]. This question was considered important to answer because widespread antibiotic use in hospitalised cases with COVID-19 was reported in the literature at the time when there were few publications regarding co-infections and superinfections [14]. Furthermore, early guidelines for COVID management also recommended early use of antibiotics (within 1 h of presentation) in all suspected COVID-19 cases on identification of sepsis [16]. Some of the literature reviewed suggested that co-pathogens were encountered in 8% of patients with COVID-19, usually those that were more severely ill and those who died, but this appeared to be mainly superinfections in the later stage of illness rather than initial co-infection [10, 13, 14].

One way of describing infections is to divide them into community-acquired versus hospital-acquired. The US Centers for Disease Control and Prevention (CDC) 1988 guideline definition is the most widely accepted one [17] and indicates that infections identified more than 48 h after hospital admission should be referred to as hospital-acquired and that those within 48 h of admission as community-acquired [17, 18]. However, not all patients with infections are either admitted to hospital and others are not admitted at the start of their infection, but some time later. Another description may be co-infections and secondary/superinfections. The CDC defines superinfections as “an infection following a previous infection especially when caused by microorganisms that are resistant or have become resistant to the antibiotics used earlier”, while a co-infection is one occurring concurrently with the initial infection, the difference being purely temporal [19, 20]. These are the definitions that will be used in the current manuscript. Bengoechea and Bamford indicated that they envisioned three non-mutually exclusive scenarios of bacterial/SARS-CoV-2 co-infection; namely, secondary SARS-CoV-2 infection following initial bacterial infection or colonisation, combined viral/bacterial pneumonia, or secondary bacterial superinfection following initial SARS-CoV-2 [15]. However, as will be seen in this review there is often not a clear distinction made in the literature as to what the authors are referring to, whether true concomitant/co-infections or superinfections. One remaining issue that needs to be addressed, is that for the diagnosis of co-infections and secondary/superinfections, most commonly used are the multiplex high-throughput systems on respiratory samples [21]. The difficulty then is differentiating in a patient with a lower respiratory infection, whether the positive respiratory tract test represents carriage or true infection. For example, with regard to viral pathogens, even asymptomatic adults may have viral carriage and furthermore, significant isolation of viruses has been documented in patients undergoing mechanical ventilation for reasons other than a severe respiratory tract infection [21]. These aspects may represent a potential limitation in some of the studies that will be described. This current review aimed to evaluate the literature regarding the occurrence of co-infections and/or superinfections, particularly bacterial infections, which would require specific antibiotic therapy in their own right, in patients with COVID-19 pneumonia.

## Antimicrobial stewardship

The reason that it is important to identify whether co-infections do occur in patients with COVID-19 and whether this would justify the need for initial empiric antibiotic treatment, is due to concerns of complications and adverse events that may occur with the routine use (and overuse) of antibiotics, with subsequent development of resistant hospital-acquired, bacterial and fungal pathogens, which are contrary to antimicrobial stewardship program aims and principles [14, 15, 22–24]. Many of the pandemic viral pneumonias have similar clinical and radiological features that may make it difficult to distinguish from other common bacterial (such as pneumococcal, staphylococcal and *Klebsiella* spp.), viral (seasonal respiratory viruses), or fungal (e.g. *Pneumocystis jirovecii*) causes of pneumonia, as well as tuberculosis, and make it difficult to determine who should, or should not, get antibiotics, in addition to treatment for COVID infection, especially without additional testing [24].

It has been recognised that the COVID pandemic has had significant implications for antimicrobial resistance, both good and bad [15, 22]. Some of the aspects that may positively affect antimicrobial resistance are social distancing with limitations of contact between people, the wearing of facemasks and the recommendations on regular hand washing, as well as the isolation of infected cases with subsequent careful sterilisation of their environment [22]. The downside may be the overuse of antibiotics, if they are used routinely, which is reported to be very common, as well as the use of antimicrobials as “repurposed drugs” to treat the COVID infection itself even without co-infection [15, 23–25]. One large study from the US documented that early empiric antibiotic therapy was used in 56.6% (965/1705) patients hospitalised with COVID-19, whereas only 3.5% (59/1705) of patients had a confirmed community-onset bacterial co-infection [26]. Attempts have been made to try and objectively determine the presence of co-infections (as well as superinfections) in patients with COVID infection on hospital admission for more targeted initial antibiotic use, and to this end, it has been suggested that procalcitonin, in particular, may be a useful biomarker [27]. In the very early study from Wuhan, most patients with COVID infection had normal procalcitonin levels on admission, but four patients subsequently developed secondary infections in the ICU and three of these had procalcitonin levels > 0.5 ng/ml [10].

## Clinical data on co-infections with COVID

Following the initial studies identifying the novel coronavirus causing COVID-19 infection, and identifying the demographic, clinical, and laboratory features, as well as markers of severity and outcome, a number of additional aspects of these infections were investigated, among these the occurrence of co-infections and superinfections.

### Letters to the editor, including research letters

Cox and colleagues [28] and Zhou and colleagues [29] noted, in letters to the editors of the respective journals, that knowledge of co-infections and/or secondary infections in COVID-19 patients, was poor, but was essential to be characterised since it would have an evidence-based impact on the management and treatment of COVID cases, could save lives, particularly among those with severe infection, as well as furthering antimicrobial stewardship initiatives. While agreeing with these authors, another investigator reviewed microbiology results of patients with COVID-19 infection admitted to Whiston Hospital in the United Kingdom (UK), and concluded that bacterial co-infections were uncommon, as opposed to what happened in patients with influenza [30]. Other investigators provided initial case studies of co-infection of single or few patients with SARS-CoV-2 infection with influenza virus [31–33], and with common bacterial pathogens [34, 35]. Furthermore, relatively small studies were reported from China [36], France [37], and the United States [38], documenting co-infections in cases with COVID-19; the first documented the occurrence of co-infections with respiratory viruses, sometimes multiple, the second documented bacterial co-infections, sometimes multiple, and the third documented both viral and so-called “atypical pathogen” coinfections, sometimes multiple. In addition, another study from France, in patients with SARS-CoV-2 infection requiring mechanical ventilation for acute respiratory distress syndrome, documented that early coinfection (samples taken within 24 h after intubation) with bacterial pathogens occurred in 13 (27.7%) of the patients, with co-infection with multiple pathogens in five patients (10.6%) [39]. However, in a number of the studies described above, patients had been symptomatic for several days before hospital admission and spent several hours in hospital before intubation, so these cannot truly be labelled co-infections and may well be superinfections. Lastly, a prospective study of patients admitted to a Spanish ICU, reported both early infections (on admission or within 48 h of admission to the ICU) and later infections in 92 patients [40]. Overall, 32 microbial isolates were found within 48 h in 24 patients (26%, 24/92), most commonly *S. aureus, S. pneumoniae*, and *H. influenzae.* In some of these patients *P. aeruginosa* was isolated, but these patients had had a longer hospital stay before ICU admission (median 9 days), than that of the general group (median 3 days). However, by strict definition all of these cases would have been labelled as hospital-acquired infections. Additionally, in that study 125 microbial isolates were found in 43 patients during their ICU stay, and most were typical bacteria and fungi associated with nosocomial infections. Overall, 90% of patients received at least one antibiotic for a median of 6 days, and 12% received antifungal agents. Interestingly, the procalcitonin levels were significantly lower in those that did not appear to have a secondary infection (median 0.4 ng/ml (IQR 0.1–0.4)) versus those with an apparent infection (median 1.2 ng/ml (IQR 0.3–2.6)).

### Case reports

A number of case reports, some with additional literature reviews, have documented the occurrence of co-infection with COVID-19 and influenza [41–43], with other viral respiratory pathogens [44], and with common bacterial respiratory pathogens, including *Streptococcus pneumoniae* [45–48]. Ozaras and colleagues, reporting six patients with COVID-19, co-infected with influenza, noted that their cases were mild to moderate in severity, that the reports of this in the literature were sparse and that unless patients were specifically screened for co-infections, these would remain undiagnosed and, therefore, underestimated [41]. Some of these case reports documented co-infection with the pneumococcus, either alone or together with other pathogens [46, 47]. Cucchiari and colleagues [47] reported a series of five cases of apparent COVID-19 infection (three confirmed by PCR and two suspected and treated as such, as per protocol), with “superinfection” (as described in the article title) with the pneumococcus. It appears from reviewing the article that these were likely to be co-infecting pathogens in COVID-19 patients presenting with concomitant pneumococcal infection, with all the pneumococcal diagnoses made on initial urine testing. Importantly, procalcitonin did not appear to be sensitive enough to detect the associated bacterial infection. Antibiotics were initiated promptly and all patients survived.

### Case series

A number of mostly retrospective studies have been reported from China [49–54], the US [55–57], the UK [58, 59], Spain [60], France [61] and Iran [62], that investigated what the authors call “co-infections” in patients with COVID-19 infection. However, when reviewing several of these studies, it is not entirely clear that these were true co-infections, rather than superinfections, as defined above. This was because of an unclear indication in some studies as to when the additional microbiological specimens were taken and/or when the co-pathogens were isolated, and the indication in other studies that these infections were noted as occurring during hospitalisation. Furthermore, the nature of the pathogens isolated in some studies, are more compatible with these being hospital-acquired, rather than community-acquired, infections. In addition, many of these studies are not directly comparable because of differences in the types of specimens harvested, as well as differences in the panel of pathogens investigated, as well as the type of testing performed. However, these studies are included in this review, for completeness, below.

In the first of these studies by Zhu and colleagues, 257 laboratory-confirmed adult and child COVID-19 cases were recruited, the diagnosis was reconfirmed by real-time PCR, and specimens were tested for 39 respiratory pathogens [49]. Overall, 24 respiratory pathogens were found among the patients, 242 (94.2%) of whom were co-infected with one or more pathogens, including 11 different bacteria, nine viruses and four fungi. While it is clear that not all these infections were truly co-infections, as defined above, most had been documented within 1–4 days of onset of COVID-19 disease; however, follow-up did extend beyond this time and pathogens isolated did vary according to time of onset of the co-infection. In addition, bacterial pathogens, common in community-acquired infections, were dominant in this cohort, although nosocomial-type pathogens were also seen, albeit less frequently. The most common bacterial isolate was *S. pneumoniae*, followed by *K. pneumoniae* and *Haemophilus influenzae*. Multiple co-infections were also common. There were also differences in the number and types of pathogens isolated based on the severity of infection. With regard to the percentage of co-infections documented, this study appears to be somewhat of an outlier.

In the study by Zhang and colleagues, although these infections were called co-infections, they appear to be hospital-acquired infections and were caused by bacteria more commonly noted in these situations (*Acinetobacter baumannii*, *Escherichia coli*, *Pseudomonas aeruginosa* and *Enterococcus*). These authors noted that there was a higher rate of co-infections with bacteria and fungi in those patients with severe COVID-19 infections, who were also more likely to suffer complications and death [50]. Lv and colleagues did a retrospective cohort study and among other factors, documented co-infections in COVID cases by evaluating the results of additional nasopharyngeal swabs taken on admission for viral isolation, as well as sputum for identification of 13 respiratory pathogens, including respiratory viruses and “atypical pathogens”, blood cultures and bronchoalveolar lavage fluid for bacterial and fungal isolation [51]. It is not clear when the latter three types of samples were taken, but it would appear that these were taken some time later during hospitalisation. There was variable documentation of co-pathogens from the different samples, with respiratory viruses and *Mycoplasma pneumoniae* documented from sputum specimens, with more common nosocomial pathogens, such as *A. baumanii, Escherichia coli, Staphylococcus haemolyticus, Pseudomonas aeruginosa, Enterococcus faecium,* and *Candida* spp., being isolated from BAL or blood*.* The findings of co-pathogens alone, or together with a low lymphocyte count, or together with the low lymphocyte count in addition to elevated levels of D-dimers, were shown, on stepwise multivariate regression analysis, to be associated with severity of COVID-19 infection [51]. Similarly, the study by Chen and colleagues in COVID-19 cases indicated that additional microbiological testing from throat swab testing, sputum or endotracheal aspirates was obtained at hospital admission for determination of viral, bacterial and fungal infections, as appropriate [52]. Although no additional viruses were documented in any of the patients, the bacteria and fungi isolated were more closely related to those nosocomial pathogens described above. The additional studies from China confirmed indicated the occurrence of co-pathogens in patients with SARS-CoV-2 infection [53, 54].

Nowack and colleagues, in a study from the US, documented that co-infections with other respiratory viruses appeared to be uncommon [55]. These authors noted that infections with rhinovirus, enterovirus, and influenza were particularly uncommon, and with these low numbers of additional pathogens, they were not able to determine if co-infections were associated with severity of illness or a modified disease course. Another two studies from the US documented a variety of co-infections in COVID-19 patients; these studies were not restricted to respiratory infections alone [56, 57]. The former study documented bacterial co-infection in 46 (19%) of patients, of which the genitourinary tract was the most frequent site (57% of infections), followed by skin infections (10%) and then respiratory infections (8%). Concomitant bacterial infections were independently associated with in-hospital mortality. Overall 67% of patients received an antibiotic, but 72% of them did not have a secondary bacterial infection. Similarly, Nori and colleagues observed the occurrence of bacterial or fungal infections in COVID-19 patients admitted in the US [57]. Overall, 91 (60%) had positive respiratory cultures, 82 (54%) of patients had positive blood cultures and 21 (14%) had both. It is not clear when the additional microbiology specimens were taken, but it would appear that these, at least, included nosocomial infections, and the spectrum of pathogens found, particularly in the respiratory co-infections, is more like those of the nosocomial pathogens described above.

Similarly, it is clear in the study from the UK that the additional microbiological specimens had been taken at any time during the hospitalisation of COVID-19 cases, and, as such, many of the isolates would have included nosocomial pathogens [58]. However, the authors did classify the time of isolation of the co-pathogens as occurring early (less than 120 h from admission; which they described as likely community-acquired pathogen), or late infection (more than 120 h; which they described as likely nosocomial pathogen). However, these time cut-off points do not match the widely accepted CDC definition of a 48 h cut-off, described above [17]. However, the UK authors indicated that this was the local definition for hospital-acquired pneumonia (> 5 days) and was agreed upon to be used by the study team. The authors confirmed a low rate of early phase COVID-19 co-infection with bacteria, and there was no evidence of fungal infections. The authors concluded, similar to that described in the guidelines above that if antibacterial agents are considered indicated, they should be prescribed in line with local guidelines, and if no evidence of bacterial co-infection is found after 48–72 h, consideration should be given to stopping them [58]. Another UK study suggested that the risk of testing positive for SARS-CoV-2 was 68% lower among influenza positive cases, suggesting possible competition between the two viruses, but also confirmed that patients with co-infection had a risk of death of 5.92 (95% CI 3,21–10.91), compared with either infection alone, suggesting possible synergistic effects in co-infected individuals [59]. Garcia-Vidal and colleagues also undertook an observational study to document, among other factors, co-infections, and super-infections in hospitalised patients with COVID-19 [60]. The additional bacterial, viral, and fungal investigations on blood, sterile fluids, sputum, and other samples had been taken at the time of hospital admission, as requested by the attending physician. The different types of infection (e.g. respiratory, bloodstream, urinary infection) had strict definitions for this study and the clinically indicated infections were characterised as co-infections or super-infections, with community-acquired infections being defined as those on admission or within 24 h of admission. Overall, 31 of 989 (3.1%) patients had 37 community-acquired co-infections. Furthermore, 30 community-acquired bacterial pneumonias were documented in 21 (2.1%) patients at COVID-19 diagnosis. Two of these co-infections were with different bacteria (*S. pneumoniae* [one associated with *Moraxella catarrhalis*] and *S. aureus* [one associated with *Haemophilus influenzae*] were the most common bacterial pathogens). Viral community-acquired infections occurred in 7/989 (0.6%) patients of whom 1 presented with bacterial co-infection, as well (4 cases of influenza A, 1 of influenza B, 1 RSV, and 1 herpetic disease). Patients with community-acquired infections were admitted to ICU more frequently.

The study by Contou and colleagues was a retrospective study of adults in an intensive care unit setting investigating all microbiological studies performed in COVID-19 cases within the first 48 h of ICU admission and noted that the bacterial co-infection rate was 28% mostly related to *S. aureus, H. influenzae, S. pneumoniae,* and *Enterobacteriaceae* [61]. The median time between hospital and ICU admission of the patients was 1 (0–4) days and 30% of the 93 patients were admitted to ICU 48 h or more following hospital admission; therefore, while some of these infections may have been true co-infections, others would have been nosocomial-type superinfections. The authors suggested that their study confirms the need to institute third-generation cephalosporin therapy in confirmed COVID-19 cases in their ICU with rapid de-escalation as soon as possible. A further very small study among COVID-19 patients in ICU in Iran reported bacterial co-infection in all cases, most commonly due to *Acinetobacter baumanii* and possibly really representing superinfections [62].

Additional, apparently prospective, studies were reported from China and the United States. In one study, the authors recruited 68 patients with acute COVID-19 infection, confirmed by PCR, in Qinqdao and Wuhan, and performed indirect fluorescence testing for specific IgM antibodies in acute phase serum for detection of common respiratory pathogens [63]. From Qingdao, 24 (80.00%) of the patients had IgM antibodies against at least one respiratory pathogen, whereas only one (2.63%) patient in Wuhan had a positive result. The most common pathogens in the former cases were influenza viruses A and B, followed by *Mycoplasma pneumoniae* and *Legionella pneumophila.* Interestingly, the co-infection rate for CAP cases in Qingdao was only 20.9%.

Three additional studies were performed in the US, one among adult patients who because of symptoms, presented to 790 different types of facilities throughout the US for testing, of whom some tested SARS-CoV-2-positive and others tested SARS-CoV-2-negative [64]; another in the frail elderly in nursing homes and assisted living facilities [65]; and a third in hospitalised adults [66]. The first study documented that rates of  infections with non-SARS-CoV-2 pathogens were higher in SARS-CoV-2-positive versus –negative cases (86% versus 76%; *p* < 0.0001), that among the bacterial pathogens in both groups, *K. pneumoniae* and *M. catarrhalis* were most common, and that advanced age and nursing home status were associated with higher bacterial co-infection rates in SARS-CoV-2-positive cases [64]. Wolfe and colleagues noted essentially similar findings in their study, and the common bacterial co-pathogens were *S. aureus* and *K. pneumoniae*, in 55.8 and 40.1% of SARS-CoV-2-positive patients, respectively [65]. In contrast, in 289 adults hospitalised in the US for SARS-CoV-2 infection, 48 (16.6%) had co-infections (defined as co-pathogens detected within 72 h of confirmed SARS-CoV-2 infection) and 25 (8.7%) of these were bacterial respiratory co-infections [66]. The patients with bacterial co-infections had higher WCC, LDH, CRP, procalcitonin, and IL-6 levels. In addition, ICU admission (84.0% vs. 31.8%), mechanical ventilation (72.0% vs. 23.9%), and in-hospital mortality (45.0% vs. 9.8%) were higher in those with bacterial co-infection than those without. Using Cox proportional hazards regression and following adjustment for age, ICU admission, mechanical ventilation, corticosteroid administration, and pre-existing comorbidities, patients with bacterial co-infections had an increased risk of in-hospital mortality (adjusted HR 3.37; 95% CI 1.39–8.16; *p* = 0.007). Subsequent infections (defined as co-pathogens identified > 72 h after confirmed SARS-CoV-2 infection) were uncommon (21 infections in 16 (5.5%) patients).

### Co-infections in children with COVID

Relatively few studies have been undertaken describing co-infections in children with COVID-19 infection [67–70]. Nevertheless, what has been undertaken suggests that paediatric patients with COVID-19 infection present with epidemiological, clinical, and radiological characteristics that are distinct from adults. Furthermore, co-infections are more common than in adults, (approaching 50% co-infections with common respiratory pathogens) and the pneumococcus plays an important role in the development of lower respiratory tract infections associated with paediatric COVID infection. Lastly, elevated procalcitonin, and a consolidation with a surrounding halo sign, may be more common than in adults, possibly representing a typical sign in paediatric patients [67–70].

### Co-infection with other respiratory pathogens

#### Tuberculosis

There have also been reports of co-infection in patients with COVID-19 involving other respiratory pathogens, which may be more common in some regions than in others. The first of these is tuberculosis (TB). Chen and colleagues described both active and latent TB as being a risk factor for COVID-19 infection, in an observational case-control study from Shenyang, China [71]. TB diagnosis was based on an interferon-gamma release assay on peripheral blood. Not only were patients with active or latent TB more susceptible, but the symptom progression of the COVID infection was more rapid and more severe. While suggesting that these findings needed to be confirmed in much larger studies, these authors suggested that all patients with COVID-19 infection should be tested for TB. Subsequently, case reports began emerging that documented TB in COVID-19 cases on the basis of both positive smear and the Gene-Xpert MTB/RIF sputum assay [72]. Thereafter a case-control study of 49 cases, which was a global cohort of current or former TB patients (with post-TB sequelae), was published, including patients from eight countries and three continents [73]. The authors divided patients into cases with TB before COVID-19, those with COVID-19 followed by TB, and those in whom these two infections occurred in the same week. There was some discussion in the literature about the interpretation of the findings, particularly regarding the timing, with the suggestion that since TB has a chronic course, while COVID was an acute illness, this co-infection may be purely incidental; however, there was concern about the high mortality of that study of 12.3% in the cases with apparent co-infections, which is higher than that for COVID alone [74, 75]. There was also a concern though, aside from these comments, that these co-infections, even if co-incidental, may nevertheless be an issue in countries with high TB (and post-TB sequelae) burdens and that both infections could have a significant, synergistic social and economic impact worldwide [74]. A further concern with regard to TB is the potential impact that the COVID pandemic may have on national programs for eradication of diseases such as TB, with recognition of the important need to continue and even strengthen these national programs and encourage people to continue to access healthcare for timely diagnosis and treatment of TB, as required [76, 77].

#### Atypical pathogens

Case reports of SARS-CoV-2 and *Legionella* coinfection have been described [78]. Oliva and co-workers [79] reported a case series of SARS-CoV-2 with *Chlamydia* or *Mycoplasma* infections and Nicolson and colleagues [80] reviewed the evidence for whether these infections are linked to progression of the COVID-19 disease and its lethal outcome.

#### Fungi

Also, reports have emerged, documenting cases with coronavirus disease 2019 associated with *Pneumocystis jirovecii* pneumonia, either simultaneously, or within a few days of each diagnosis, which represents a particular diagnostic dilemma, especially in people living with HIV unless routinely tested for [81, 82], and with other fungal infections [83].

#### Other

Measles has been described in association with SARS-CoV-2 in a front-line healthcare worker [84], as well as the association of dengue with SARS-CoV-2 infection [85]. Lastly, Abdoli [86] expressed concerns that helminthic co-infection may increase morbidity and mortality in COVID-19 due to their suppressive effect on the immune response.

#### Review articles

Lastly, several reviews, some with meta-analyses, have been published describing the occurrence of co-infections and secondary infections in patients with COVID-19 infections [87–93]. Several of these authors reported that the rate of co-infections was low, with bacterial and fungal infections occurring in 8% of hospital admissions in one study [88], and bacterial, fungal, and viral infections in 7% (95% CI 3–12%, *n* = 2183, I^2^ = 92.2%) of hospitalised cases in another study, with a higher proportion of ICU cases having co-infections than in mixed ward/ICU settings (14, 95% CI5–26, I^2^ = 74.7% versus 4, 95% CI 1–9, I^2^ = 91.7%) [86]. Both studies appear to have been conducted in all patients, including both adults and children, and it appears that both true co-infections and secondary infections were included. Langford and colleagues performed a living rapid review and meta-analysis of bacterial co-infection and secondary infection, using the CDC definition of such cases [90]. Bacterial co-infections were reported in 3.5% (95% CI, 0.6 to 6.5%) of patients on admission with COVID and secondary infections were reported in 15.5% (95% CI, 10.9 to 20.1%); while the overall rate of bacterial infections was 7.1% (95% CI, 4.6 to 13.8) and more common in critically ill patients (8.1%; 95% CI, 2–3 to 13.8%). The authors attribute the possible reasons for differences in the reported bacterial co-infections and superinfections as being due to regional variations in patient populations, their access to care, and infection prevention and control measures implemented. Most of the studies reported high rates of antibiotic use, being 71.3%; 95% CI, 57.1 to 85.5% in the latter study, mostly broad spectrum, and suggested that most patients may not require them. This may also have had impacted on the documentation of additional bacterial infections.

Lai and colleagues did an extensive literature review of both co-infections and secondary infections with viruses, bacteria and fungi in patients with COVID-19 [91]. There was no clear mention of how these cases were characterised, and presumably, was based on the individual authors’ consideration. They noted that the studies were all observational, cross sectional studies and that there was considerable variation in the reporting of co-infections in the different studies. However, they did report that it could be as high as 50% among non-survivors. There was a range of viral and bacterial pathogens noted, many of which appear to be the common, community-acquired pathogens, but some studies reported pathogens that more clearly appear to be nosocomial pathogens. Furthermore, the authors noted that clinical, radiological, and routine laboratory data could not distinguish between co-infecting pathogens and COVID-19. The authors correctly concluded that future large-scale well-designed, prospective studies needed to be conducted to answer the questions regarding the true prevalence of COVID-19 co-infection, the risks of such infections, the microbiological causes of such infections, and their impact on patient outcomes. Only, thereafter, can informed decisions be made regarding empirical antibiotic use in suspected cases of COVID-19 infection. Anthony and coworkers also recognised, particularly because of the similarity of the presentation of COVID-19 and influenza, that coinfection rates may not be low, but rather underreported [92].

Lastly, while it would have been ideal in the current review to grade the evidence for the occurrence of co-infections and superinfections according to the quality and frequency of the additional testing, this would not have been possible. Many of the studies are small, often they are retrospective, clear indication of when the additional testing was undertaken was usually not evident and several of the studies were not primarily set out to document these infections. All these issues, as well as the issue regarding differentiating carriage from true infections, with the additional testing, need to be appropriately and comprehensively addressed in future studies.

## Mechanisms of viral-bacterial interactions

The next section of this review will describe the mechanism(s) most likely associated with the occurrence of microbial co-infections and superinfections, starting initially with the apparent synergistic effects of viral-bacterial interactions. A significant amount of information in the literature regarding these effects relates to the interaction between the influenza virus and bacterial pathogens, in particular, *S. pneumoniae.* This is followed by a review of what is known about the mechanisms of these interactions, as well as mechanisms that are suggested, regarding the interactions of SARS-Cov-2 with other pathogens. The latter will include an overview of the potential role of activated platelets, and their immunosuppressive effects, in enhancing the risk of SARS-CoV-2 co-infections and superinfections.

When reviewing the data presented below, the reader will recognize that much of what is described in the literature, relates to the occurrence of viral infections, followed some time later by, most commonly, bacterial infections, and, therefore, that these mechanisms described relate predominantly to superinfections rather than true co-infections, as we have defined in the current review.

### Synergistic effects of influenza virus and *S. pneumoniae*

Secondary bacterial pneumonia, particularly due to the pneumococcus, following influenza epidemics and pandemics, as an important cause of excess mortality, initially suggested in studies from the 1918 influenza pandemic, was subsequently confirmed during the 2009, H1N1, influenza pandemic [94]. Studies have reported that the mechanisms may include; i) destruction of the respiratory epithelium and exposure of the basement membrane [95], and, ii) upregulation of molecules that bacteria use as receptors (especially due to the viral neuraminidase activity) [95], both of which increase bacterial adherence to the respiratory epithelium, as well as, iii) impaired function of immune cells, including neutrophils and macrophages, the latter affected by the release of interferon-gamma produced during T-cell responses to influenza, that impairs clearance of pneumococci for the lung by alveolar macrophages [96]. Through the mechanisms described above, influenza A virus infection has been shown to facilitate pneumococcal colonization, transmission, and active disease [97], although, as shown by others, this appears to be independent of the upregulation of the platelet-activating receptor (PAF-R) [98]. In experimental models, the survival of non-lethal exposure of mice to influenza followed 7 days later by non-lethal pneumococcal exposure resulted in 100% mortality; however, when the order was reduced there was protection from influenza and improved survival [95, 98]. Additional studies have highlighted the important role that *S. pneumoniae* played in the excess mortality of patients during the 1918 and the 2009 influenza pandemics [94, 99, 100].

### Molecular pathogenesis of secondary bacterial infection in association with SARS-CoV-2

A number of investigators recently reviewed the proposed mechanisms by which viral infections, and particularly SARS-Co-V-2, may predispose to concomitant and subsequent bacterial infections [15, 101]. They emphasised that the damage that viruses cause to the respiratory epithelium, as well as their effects on innate and adaptive immunity, antagonising IFN responses that enhance bacterial adherence, colonisation, growth, and invasion into healthy sites in the respiratory tract, are important mechanisms [15, 101]. They then translate many of these mechanisms into what is known with regard to the SARS-CoV-2 virus, or indicate putative mechanisms.

Mirzaei and colleagues [87] provide a very valuable overview of bacterial coinfections with viruses, in general, and SARS-CoV-2, in particular, reviewing in detail possible putative mechanisms by which viruses may predispose to bacterial coinfection but also postulating the mechanisms by which bacterial coinfection with SARS-CoV-2 occurs, and providing functional suggestions for both the management and control of them.

Manna and colleagues further indicate that SARS-CoV-2 is similar to SARS-Co-V, which has previously been reported to regulate immune function-related gene expression in human monocytes. Immune-related gene expression suggested that SARS-CoV-2 downregulates IFN-α/β-inducible and cathepsin/proteasome genes, while differentially regulated genes include TLR/TLR-signalling, cytokine/cytokine receptor-related, chemokine/chemokine receptor-related, lysosome-related, MHC/chaperon-related and fibrosis-related genes. In a separate study, SARS-CoV was also reported to suppress type 1, IFN production, an activity that compromises alveolar macrophage recruitment and function. Downregulation and differential regulation of immune genes are mechanisms that may create a positive environment for establishment of secondary bacterial infections [101], favouring bacterial attachment to host structural cells and pro-inflammatory environment conducive to suppression of anti-bacterial host defences. In addition, Bogeochea and Bamford [15] also question whether SARS-CoV-2 may perturb gut homeostasis. Since the importance of the gut-lung axis in controlling bacterial pneumonia is well established, disturbance of the gut microbiota may well be a mechanism that may potentially affect the disease outcomes in patients with severe COVID-19 infection, including predisposing to secondary lung infections [15].

Lastly, Golda and colleagues documented that the human coronavirus NL63 enhanced adherence of *S. pneumoniae* to virus-infected cell lines, and in fully differentiated primary human airway epithelial cell cultures [102]. Interestingly, this enhanced binding correlated with an increased expression of the platelet-activating factor receptor, but much as Diavotopoulos et al. described above [97], a detailed evaluation of the bacterial-PAF-R interaction suggested limited importance of this mechanism.

### Platelets in the pathogenesis of SARS-CoV-2 secondary infections

The preceding clinical overview has highlighted the complexity of distinguishing between co-infection and super-infection following hospital admission of patients with severe COVID-19 infection. Given the identities of several of the common causative pathogens together with poor responsiveness to antimicrobial chemotherapy and unfavourable clinical outcomes, super-infection secondary to severe SARS-CoV-2-associated immunosuppression [103, 104] exacerbated in many cases by immunosenescence, seems prominent. This contention is supported by the additional risks posed by the contribution of inappropriate use of antibiotics administered to patients with less severe disease to the emergence of multidrug-resistant microbial pathogens in the hospital environment together with the fact that those with severe COVID-19 and associated immunosuppression must also endure prolonged hospital stays, often necessitating mechanical ventilation in the ICU setting, posing the potential hazard of nosocomial infection.

In addition, intense immunosuppression associated with SARS-CoV-2 infection may also trigger the activation of quiescent, biofilm-encased airway pathogens such as the pneumococcus, *H. influenzae* and *S. aureus.*

#### Platelet-driven immunosuppression

The human platelet has been increasingly recognized as being a key player in orchestrating the excessive COVID-19-related systemic inflammation that drives not only generalized immunosuppression, but also the development of acute respiratory distress syndrome (ARDS) and cardiac dysfunction that severely complicates this acute viral disease [105–108]. In this context, platelets have been reported to express angiotensin-converting enzyme 2 (ACE2), presumably derived from megakaryocytes, that interacts with the spike protein of SARS-CoV-2, resulting in platelet activation [109]. Indices of platelet activation driven by this mechanism include cellular aggregation, upregulation of expression of the adhesion molecule, CD62P (P-selectin), activation of the integrin, GP11b/111a, mobilization of both α-granule and dense granules, and platelet spreading [109]. These pro-inflammatory/pro-thrombotic activities of platelets detected in vitro correlated with increased mean platelet volume and thrombocytopenia [109]. However, others were unable to detect the presence of either mRNA encoding ACE2 or the protein per se in platelets from COVID-19 patients [105], suggesting the existence of alternative mechanisms of SARS-CoV-2 platelet activation. These most likely involve the recognition of viral single-stranded RNA by platelet Toll-like receptor 7 (TLR7) during the viraemic phase of the disease, which may precede the onset of symptoms [110]. Although potentially protective in early-stage disease [111–113], uncontrolled systemic hyperactivation of platelets results in inflammation-triggered immunosuppression and microvascular occlusion. Prominent mechanisms of platelet-driven systemic immunosuppression in this setting include the following:

• Upregulation of expression of CD62P, which, in turn, interacts with its counter-ligand, P-selectin glycoprotein ligand-1 (PSGL-1), expressed on neutrophils, monocytes, T lymphocytes and vascular endothelium [105, 114];

• Intravascular formation of heterotypic aggregates between platelets with neutrophils, monocytes, and T cells, resulting in inappropriate leukocyte activation and microvascular occlusion [105, 106];

• Augmentation of formation of platelet/neutrophil and platelet/monocyte aggregates via binding of cell surface-expressed platelet factor 4 (PF4, also known as the chemokine, CXCL4) with the leukocyte integrin, CR3 [115];

• Mobilization of platelet intracellular granules resulting in the release of the neutrophil/monocyte-activating pro-inflammatory chemokines, CXCL4 (PF4), CXCL8 (IL-8) and RANTES [107], as well as the immunosuppressive cytokine, transforming growth factor (TGF)-β [116];

• TGF-β-mediated polarization [116, 117] of immunosuppressive M2-like macrophages [118, 119], regulatory T cells (Tregs) [104, 120] and myeloid-derived suppressor cells (MDSCs) [121, 122];

• Release of pro-inflammatory/pro-thrombotic, cytosolic group box 1 (HMGB1) protein [123];

• Given the presence of both spliced mRNA encoding pro-IL-1β and the NOD-like receptor protein 3 (NLRP3) inflammasome in platelets, it seems likely that triggering of TLR7 will also lead to the synthesis, proteolytic modification, and release of biologically-active pro-inflammatory IL-1β [124].

Other potential mechanisms of TGF-β-mediated immunosuppression include platelet-derived TGF-β-mediated production of IL-6 by hepatic endothelial cells [125], which, in turn, promotes increased production of thrombopoietin [126], driving thrombocytosis and platelet activation.

These and other mechanisms, such as those driven by platelet-activated neutrophils are likely to underpin the intense immunosuppression associated with severe COVOD-19 [104].

#### Platelets and NETosis

In addition to the aforementioned mechanisms of immunosuppression, platelets and their mediators of inflammation, specifically reactive oxygen species, HMGB1, IL-8, and CD62P are also important drivers of the formation of neutrophil extracellular traps (NETs) which are believed to be major contributors to the pathogenesis of SARS-CoV-2-associated ARDs and cardiac damage [127–134]. Notwithstanding key involvement in intravascular and intrapulmonary obstruction [134, 135], the histone components of NETs, as well as various granule-derived proteinases, are also potent cytotoxins for epithelium and vascular endothelium [133, 136, 137]. In addition, NET-derived histone- and proteinase-mediated cytotoxic effects on epithelial and endothelial cells following infection with the human coronavirus NL63, as well as SARS-CoV-2, the influenza virus, and other respiratory viruses are probable major contributors to the development of secondary and super-bacterial infections. This results from several mechanisms, including: i) exposure of receptors for bacterial adhesins following injury to epithelial and endothelial cells; ii) release of dormant potentially pathogenic intracellular pathogens from these cells; and iii) via facilitation of extrapulmonary dissemination of bacterial pathogens [101, 102, 138].

Given the prevailing uncertainty surrounding the involvement of the “cytokine storm” in the pathophysiology of COVID-19 [139, 140], targeting of platelets in particular, as well as neutrophils, appears to represent a potentially useful strategy to counter COVID-19-associated immunosuppression.

## How to treat CAP in the COVID-19 era

This manuscript is a detailed description of co-infections and secondary infections in patients with COVID-19 infection, which clearly do occur, and which are associated with severe disease and associated poor outcome. However, in the literature reviewed there is often not a clear delineation made by the authors between co-infections and secondary/superinfections. In the most recent preprint of a systematic review and meta-analysis, the authors attempted to dissect out co-infections from superinfections [141]. They defined co-infection as the recovery of other respiratory pathogens in patients with SARS-CoV-2 infection at the time of a SARS-CoV-2 infection diagnosis and superinfection as the subsequent recovery of other respiratory pathogens during care for patients infected with SARS-CoV-2. Doing this, they found that as many as 12% of patients with COVID-19 had co-infections and as many as 14% superinfections, the latter being associated with poor outcomes [141]. Bacterial co-infections occurred in 4% (95% CI: 1–8%), and superinfections in 6% (95% CI: 2–11%), viral co-infections occurred in 4% (95% CI: 2–7%) and superinfections in 2% (95% CI: 0–7%), and fungal co-infections in 4% (95% CI: 1–8%), and fungal superinfections in 4% (95% CI 0–11%). However, it is interesting to note that a recent multicentre, international study, indicated in all countries that were included, there has recently been a significant and sustained reduction in overall invasive diseases in the communities due to *S. pneumoniae, H. influenzae,* and *N. meningitidis,* which was attributed to COVID-19 containment measures [142].

Nevertheless, the question remains as to what should be done with antibiotic therapy in the time of COVID-19. Unfortunately, the recent update of guidelines for the management of CAP, such as that of the IDSA/ATS in the US [5], occurred before the outbreak of COVID-19 infection, and so they do not contain information about management of CAP in the COVID era. Nevertheless, the main authors of that CAP guideline did offer an interpretation of how the guideline would apply to the management of patients with COVID-19, particularly with the concern regarding bacterial co-infections [143]. Several additional guidelines were subsequently published, such as those from the Netherlands (an evidence-based guideline [144]), the UK (the NICE guideline [145]), and South Africa (from the National Institute for Communicable Diseases) [146], as well as expert recommendations [147]. These either contained, among other issues, antibiotic recommendations for treatment of co-infections and secondary infections with COVID-19 infection or concentrated purely on antibacterial therapy.

Metlay and Waterer [143] offered recommendations regarding CAP management in the COVID-19 era, indicating the following; i) Empiric antibiotic therapy is recommended in patients with CAP, without COVID, but not all confirmed COVID cases, ii) The relevant bacterial pathogens in patients with CAP and COVID are likely to be the same as in patients with CAP alone and, therefore, if antibiotics are to be used they should be the same, iii), testing sputum and blood for bacterial pathogens is most useful when there is a concern for multidrug-resistant pathogens, and iv) procalcitonin may help prevent overuse of antibiotics. The NICE guideline indicated that while it may be difficult to differentiate between COVID pneumonia and either primary or secondary bacterial pneumonia, bacterial infections are more likely if patients become rapidly unwell after only a few days of symptoms, and if they do not have typical COVID-19 symptoms, but have pleuritic chest pain and purulent sputum [145]. The NICD recommendations in South Africa indicated that the initial differential diagnosis of suspected COVID-19 cases, in the correct setting, could include influenza (seasonal), conventional and “atypical” bacterial CAP, and in those with HIV, opportunistic pathogens, such as *Pneumocystis jirovecii* [146]. Furthermore, they recommended that depending on the patient, a range of microbiological investigations may need to be undertaken, and depending on the clinical and laboratory findings, patients should be treated for the appropriate condition, based on guideline recommendations [146].

The guideline from the Netherlands recommended that maximum efforts should be made in all COVID-19 cases to obtain sputum and blood for bacterial cultures and also to do urinary antigen testing [144]. Given the frequency of the occurrence of co-infections and superinfections described in the current review, this would seem to be the most logical approach to this issue. There appears to be consensus among these documents that in the presence of suspected bacterial co-infections, particularly in more severe cases, local guideline-concordant antibiotics should be commenced in patients with COVID-19, but that if all the cultures are negative (and some indicate additionally that if the procalcitonin levels are low), it may be reasonable to discontinue antibiotics [143, 144, 147]. If the microbiological results indicate the presence of a bacterial co-infection, depending on the findings, antibiotic treatment may be able to be narrowed and should be continued for 5–7 days treatment [143, 144, 147]. In the case of secondary infections, antibiotic treatment should be concordant with local guidelines for hospital-acquired or ventilator-associated infection and continued for 7 days [144, 147].

## Conclusions

It is quite clear from the review that additional infections, with various other pathogens, do occur in patients with SARS-CoV-2 infection, representing either true co-infections, or superinfections, as we defined in this review. These infections appear to be associated with the severity of COVID-19 infection and poor outcomes. It may well be difficult to differentiate which patients have co-infection/superinfection and which do not; however, the Dutch guideline recommends that a concerted effort should be made to determine if a bacterial coinfection is present on admission of a patient with COVID pneumonia, and that recommendation could be supported on the basis of the evidence presented in this review. Furthermore, many experts have provided a number of recommendations, regarding additional therapy, particularly antibiotic therapy, to guide clinicians managing patients with SARS-CoV-2 infection.

## Data Availability

The data is freely available in the literature.
